# Alternative antiretroviral monitoring strategies for HIV-infected patients in east Africa: opportunities to save more lives?

**DOI:** 10.1186/1758-2652-14-38

**Published:** 2011-07-30

**Authors:** R Scott Braithwaite, Kimberly A Nucifora, Constantin T Yiannoutsos, Beverly Musick, Sylvester Kimaiyo, Lameck Diero, Melanie C Bacon, Kara Wools-Kaloustian

**Affiliations:** 1 Section on Value and Comparative Effectiveness, Department of Medicine, New York University School of Medicine, New York, NY, USA; 2 Department of Biostatistics, Indiana University School of Medicine, Indianapolis, IN, USA; 3 Department of Medicine, Moi University School of Medicine, Eldoret, Kenya; 4 Division of AIDS, NIAID, National Institutes of Health, Bethesda, MD, USA; 5 Department of Medicine, Indiana University School of Medicine, Indianapolis, IN, USA

## Abstract

**Background:**

Updated World Health Organization guidelines have amplified debate about how resource constraints should impact monitoring strategies for HIV-infected persons on combination antiretroviral therapy (cART). We estimated the incremental benefit and cost effectiveness of alternative monitoring strategies for east Africans with known HIV infection.

**Methods:**

Using a validated HIV computer simulation based on resource-limited data (USAID and AMPATH) and circumstances (east Africa), we compared alternative monitoring strategies for HIV-infected persons newly started on cART. We evaluated clinical, immunologic and virologic monitoring strategies, including combinations and conditional logic (e.g., only perform virologic testing if immunologic testing is positive). We calculated incremental cost-effectiveness ratios (ICER) in units of cost per quality-adjusted life year (QALY), using a societal perspective and a lifetime horizon. Costs were measured in 2008 US dollars, and costs and benefits were discounted at 3%. We compared the ICER of monitoring strategies with those of other resource-constrained decisions, in particular earlier cART initiation (at CD4 counts of 350 cells/mm^3 ^rather than 200 cells/mm^3^).

**Results:**

Monitoring strategies employing routine CD4 testing without virologic testing never maximized health benefits, regardless of budget or societal willingness to pay for additional health benefits. Monitoring strategies employing virologic testing conditional upon particular CD4 results delivered the most benefit at willingness-to-pay levels similar to the cost of earlier cART initiation (approximately $2600/QALY). Monitoring strategies employing routine virologic testing alone only maximized health benefits at willingness-to-pay levels (> $4400/QALY) that greatly exceeded the ICER of earlier cART initiation.

**Conclusions:**

CD4 testing alone never maximized health benefits regardless of resource limitations. Programmes routinely performing virologic testing but deferring cART initiation may increase health benefits by reallocating monitoring resources towards earlier cART initiation.

## Background

Considerable debate exists about how resource constraints should impact laboratory monitoring for HIV-infected patients on combination antiretroviral therapy (cART) [1-6]. This lack of consensus is reflected in the equivocal language about laboratory monitoring in 2010 recommendations by the World Health Organization (WHO) [7]. WHO recommends using viral load testing every six months to detect viral replication, but only "conditionally" and "where routinely available". While WHO "strongly" recommends use of viral load "to confirm treatment failure", this recommendation is also followed by the conditional statement, "where routinely available" [7]. While this equivocal language of these recommendations may be interpreted as a pragmatic concession to resource constraints, it is important to note that no equivalent language was used in WHO recommendations for earlier cART initiation, even though this guideline is equally, if not more, impacted by resource constraints. For these reasons, the 2010 WHO recommendations are likely to amplify debate on the importance of routine viral load testing compared with other resource-constrained decisions. Published data are insufficient to guide this decision [1-6,8,9].

Published decision models have broadly suggested that laboratory monitoring delivers less favourable value than alternative resource allocations [5,10,11]. However, these models have important limitations of their own: (1) failure to consider a wide range of monitoring strategies, such as conditionally dependent strategies (e.g., only check a viral load if CD4 result meets predefined criteria); (2) failure to consider widely varying scenarios regarding number of cART regimens and their sequencing (e.g., monitoring would be expected to confer greater benefit when more regimens are available, because the information is more useful); (3) failure to compare results with other resource-constrained decisions (e.g., earlier cART initiation), asking if more lives could be saved by alternative resource expenditures; and (4) failure to use data from resource-limited settings, thus limiting their generalizability.

We have previously developed and validated a computer simulation model of HIV progression in resource-rich settings [12-15]. Our model explicitly represents the two main reasons for cART failure, genotypic resistance accumulation and non-adherence, and therefore is equipped to explore important tradeoffs involved in more versus less aggressive monitoring strategies. For example, a more aggressive monitoring strategy may result in treatment changes that support greater virologic suppression in the short term, but may exhaust available regimens in the long term. For the current report, we have redesigned and re-calibrated this model for resource-limited settings. Its design now permits consideration of widely varying monitoring strategies, including conditional strategies, under different scenarios regarding numbers and sequences of cART regimens.

## Methods

We used a computer model to simulate alternative laboratory monitoring strategies for HIV-infected patients on cART in east Africa, and to compare the value of these strategies with alternative resource allocation options, such as earlier cART initiation. This model has been previously validated by demonstrating its ability to predict clinical data describing survival, time until cART failure, and accumulation of resistance mutations in distinct observational cohorts [12-15].

**Figure 1:**
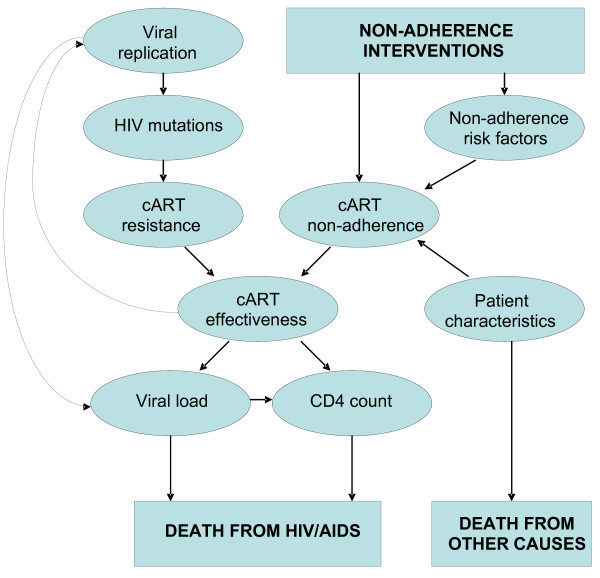
**Schematic of constructs in computer simulation**.

This simulation has been revised: (1) to allow specification of a wide variety of possible monitoring strategies; (2) to allow calibration using data from resource-limited settings; and (3) to consider a specifiable number of cART regimens or a specifiable number of drugs within each cART category (and can "run out" of regimens when intolerance and/or resistance has developed to all). The simulation is a stochastic, second-order Monte Carlo progression model that explicitly represents the two main determinates of treatment failure: accumulation of genotypic resistance and cART non-adherence and/or intolerance (Figure 1). A key advantage of this design is that it can compare tradeoffs in aggressiveness of treatment versus intensiveness of monitoring. The methods underlying the revision of this simulation and its calibration are described in more detail in the Appendix (Additional file 1), and the results of the calibration are described in Additional file 1, Figure S1.

### Analytic approach: comparison with simultaneous resource-constrained decisions

We sought to identify "efficient frontiers", defined as those strategies delivering the greatest health benefit given a plausible budget scenario [16,17]. Strategies within an efficient frontier confer the greatest benefit for a specified budget. Strategies outside this frontier are unable to deliver the greatest benefit regardless of budget, and therefore are not preferred choices regardless of available resources.

We identified efficient frontiers by calculating the incremental cost-effectiveness ratio (ICER) of each monitoring strategy. ICERs measure the additive benefit of each strategy compared with its next best alternative, and interpret this benefit together with its additive cost. The ICER compares different choices in a systematic, quantitative manner, placing them "on a level playing field", and providing a widely used quantitative measure of value. Higher ICERs (meaning a greater cost per additional benefit) are less favourable, corresponding to lower value. Lower ICERs (meaning a lower cost per additional benefit) are more favourable, corresponding to higher value. ICERs are useful for informing resource allocation decisions because reallocating resources from a numerically higher (less favourable) ICER towards a numerically lower (more favourable) ICER can increase health benefits without requiring additional resources. We performed all analyses from a societal perspective using a lifetime time horizon. All costs and benefits were measured in US dollar (USD) values for 2008 and were discounted at an annual rate of 3%. In all cases, we followed recommendations of the US Panel on Cost-Effectiveness in Health [18]. We simulated cohorts of 1,000,000, with a one-day cycle time (the minimum time interval over which patient characteristics could change).

Considerable debate exists over acceptable value "thresholds" and their appropriate variation with resource constraints [19]. To aid interpretation of our ICER results for different monitoring strategies, we used our simulation to estimate ICER results for other common, resource-constrained decisions (e.g., initiation of cART at CD4 counts of 350 cells/mm^3 ^versus 200 cells/mm^3^, and whether to make second- and third-line cART regimens routinely available).

### Base case analyses

We compared the downstream effects of alternative monitoring strategies on HIV-positive patients newly started on cART. In accord with the USAID-AMPATH experience, we assumed that the first cART regimen was nevirapine in combination with two nucleoside reverse transcriptase inhibitors. Distributions of age, sex and CD4 count at cART initiation were based on characteristics of patients enrolled in USAID-AMPATH (Table 1). We did not perform distinct analyses for women who were exposed to single-agent prophylaxis for mother to child transmission; however, the impact of nevirapine resistance was explored in a sensitivity analysis.

**Table 1:** Inputs in computer simulation

Variable	Base case	Plausible range in sensitivity analysis	Source
**Characteristics of simulated cohort**			

Age	39 (SD 9)	NA	AMPATH

CD4 count (cells/mm^3^)	126 (SD 127)	NA	AMPATH

Viral load (Log 10 units)	4.5 (SD 1)	NA	AMPATH

% Male	38%	NA	AMPATH

Initial cART regimen	Nevirapine + either ziduvidine or stavudine + other NRTI	NA	AMPATH

Second cART regimen	Boosted PI + two other NRTIs other than those in initial regimen	NA	AMPATH

**Probabilities and rates**			

Compliance with cART (proportion of doses taken as directed)	0.85	0.75-0.95	Imputed from calibration

Probability that mutation potentially causing resistance, results in resistance, NRTI or PI	0.50	Varied jointly from 0.5X to 1.5X, bounded by 0 and 1	Johnson *et al *[21]

Probability mutation potentially causing resistance, results in resistance, NNRTI	0.90	Varied jointly from 0.5X to 1.5X, bounded by 0 and 1	Johnson *et al *[21]

Probability of cross-resistance to other NRTI, given NRTI mutation conferring resistance (ziduvidine or stavudine)	1.0	Varied jointly from 0.5X to 1.5X, bounded by 0 and 1	Johnson *et al *[21]

Probability of cross-resistance to other NRTI, given NRTI mutation conferring resistance (other)	0.48	Varied jointly from 0.5X to 1.5X, bounded by 0 and 1	Johnson *et al *[21]

Probability of cross resistance to other PI, given PI mutation causing resistance	0.24	Varied jointly from 0.5X to 1.5X, bounded by 0 and 1	Johnson *et al *[21]

Probability of cross resistance to other NNRTI, given NNRTI mutation causing resistance	0.88	Varied jointly from 0.5X to 1.5X, bounded by 0 and 1	Johnson *et al *[21]

Rate of accumulating resistance mutations, per year	0.18	0.014-0.018	Braithwaite *et al *[14]

Viral load decrement with cART consisting of 2 NRTIs + efavirenz (100% adherence)	3.09	Varied jointly from -1 to +1	Braithwaite *et al *[22]

Viral load decrement with cART consisting of 2 NRTIs + nevirapine (100% adherence)	2.22	Varied jointly from -1 to +1	Braithwaite *et al *[22]

Viral load decrement with cART consisting of boosted PI (100% adherence)	2.68	Varied jointly from -1 to +1	Braithwaite *et al *[22]

Augmentation in HIV-related mortality, multiplicative	1	Varied from 0.5X to 1.5X	Assumption

Augmentation in non-HIV-related mortality, multiplicative	1	Varied from 0.5X to 1.5X	Assumption

**Utilities**			

Decrease in utility with cART	0.053	Varied jointly from -0.05 to +0.05	Braithwaite *et al *2007 [23]

Utility with CD4 < 100 cells/mm^3^	0.81	Varied jointly from -0.05 to +0.05	Freedberg *et al *1998 [24]

Utility with CD4 between 100 cells/mm^3 ^and 199 cells/mm^3^	0.87	Varied jointly from -0.05 to +0.05	Freedberg *et al *1998 [24]

Utility with CD4 200 cells/mm^3 ^and above	0.94	Varied jointly from -0.05 to +0.05	Freedberg *et al *1998 [24]

**Costs (2008 US$)**			

Cost of outpatient care, annually, without cART ($/month)	$288	Varied from 0.5X to 1.5X	AMPATH

Cost of care per hospitalization	$390	Varied from 0.5X to 1.5X	AMPATH

Cost of cART, annually, first regimen	$189	Varied from 0.5X to 1.5X	AMPATH

Cost of cART annually, second regimen	$1361	Varied from 0.5X to 1.5X	AMPATH

Cost of cART annually, third regimen	$3067	$1361 - $12,269	AMPATH, Red Book [25]

Cost of viral load test	$70.00	Varied from 0.5X to 1.5X	AMPATH

Cost of CD4 test	$11.20	Varied from 0.5X to 1.5X	AMPATH

NA: not applicable; cART: combination antiretroviral therapy; NRTI: nucleoside reverse transcriptase inhibitor; PI: protease inhibitor; NNRTI: non-nucleoside reverse transcriptase inhibitor.

We evaluated a matrix of different monitoring strategies: type of monitoring (clinical versus CD4 versus virologic versus combinations and conditional strategies); viral load threshold for switching (500, 1000, 5000, and 10,000 copies/ml); and frequency of monitoring (three, six and 12 months). Clinical monitoring was defined as evaluation by a health professional for signs and symptoms of AIDS [20]. We deliberately constructed a broad matrix of options that included some strategies that are not guideline recommended at the current time, but which might seem like plausible alternatives (for example, obtaining routine viral load without routine CD4 counts).

Because space limitations preclude the presentation of the numerous strategies that we evaluated, we focus on results from the subset of strategies on the efficient frontier. We first identified efficient frontiers for scenarios with two and three available cART regimens. We then identified the efficient frontier for a scenario that does not specify a fixed number of cART regimens, but rather allows the number of available cART regimens to vary.

We estimated outcomes of life years, quality-adjusted life years (QALY), and costs (USD). QALYs are a preference-weighted metric that incorporate both quantity and quality of life, and reflect the idea that a year of poor-quality life is valued less than a year of high-quality life [18].

### Sensitivity analyses

Because some strategies may be sufficiently close to an efficient frontier that their exclusion is solely due to statistical uncertainty (from random variation in the model), we performed sensitivity analyses in which the cost and effectiveness estimates for each strategy were varied over their 95% interpercentile range. In separate sensitivity analyses, to assess the impact of biased inputs, we varied all inputs to the model across their plausible ranges, seeking to identify whether changes in model input assumptions would lead to different strategies on the efficient frontier.

## Results

We evaluated alternative monitoring strategies: first for a treatment scenario with two available cART regimens, and then a treatment scenario with three available cART regimens. In addition, we considered a scenario that does not specify a fixed number of cART regimens, but rather allows their number to vary. For all scenarios, we assumed that cART would be started at a CD4 count of 200 cells/mm^3^, and we sought to identify strategies on the "efficient frontier" (e.g., those that could deliver the greatest health benefit given some budget or resource constraint). Monitoring strategies lying outside this "efficient frontier" cannot deliver the greatest benefit regardless of willingness to pay, and therefore should not be preferred choices.

### Scenario with two available cART regimens

**Figure 2:**
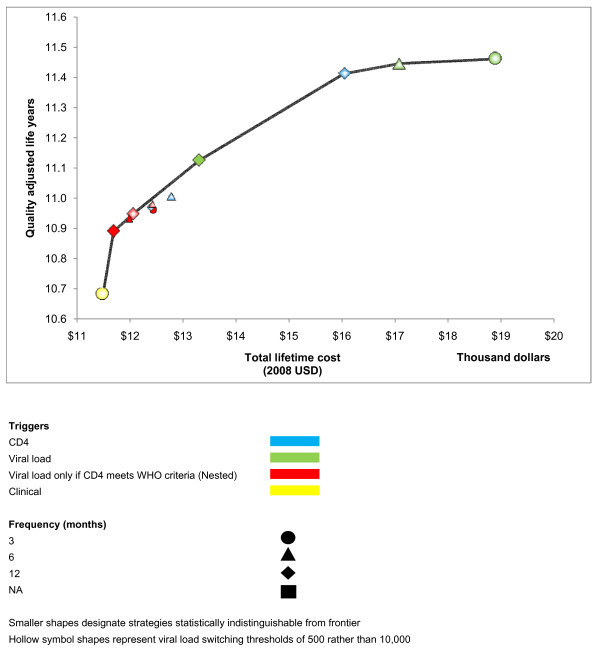
**Efficient frontier of HIV monitoring strategies assuming two cART regimens are available**.

When we explored a scenario in which two cART regimens were available (Table 2, Figure 2), no laboratory monitoring strategies employing routine CD4 monitoring alone (e.g., CD4 count every six months) were on the efficient frontier. Overall, these strategies did not offer a good use of healthcare resources, because greater benefit would be conferred by alternative strategies, regardless of a programme's budget or willingness to pay for health benefits.

**Table 2:** Value of alternative laboratory monitoring strategies compared with earlier treatment initiation, *assuming two antiretroviral regimens are available*

Monitoring strategy	Freq-uency (mo.)	Viral load threshold for switching ARV (copies/ ml)	5-year outcomes				Cost ($2008)	QALY	ICER ($/QALY)	Value com-pared with earlier treatment initiation*
							
			Mean # ARV rounds used	Mean new mut-ations	Median CD4 (cells/mm^3^)	Median HIV (log units)				
Clinical	3	N/A	1.26	1.02	270	2.66	11,490	10.681	N/A	N/A

Viral load only if CD4 meets WHO criteria†	12	10,000	1.23	1.09	270	2.70	11,691	10.890	1,000	Same or better

Viral load only if CD4 meets WHO criteria†‡	12	500	1.27	1.06	270	2.66	12,060	10.948	6,400	Worse

Viral load¶	12	10,000	1.33	1.02	277	2.69	13,308	11.125	7,100	Worse

Viral load	12	500	1.67	0.82	285	2.42	16,035	11.412	9,500	Worse

Viral load	6	500	1.69	0.81	286	2.40	17,087	11.446	30,900	Worse

Viral load	3	500	1.70	0.79	286	2.39	18,901	11.461	121,000	Worse

Mo.: months; QALY: quality-adjusted life year; ICER: incremental cost-effectiveness ratio.

* Earlier treatment initiation at CD4 of 350 cells/mm^3 ^compared with CD4 of 200 cells/mm^3^. "Better" value is indicated by a numerically lower ICER, and suggests that health benefits would be increased if resources were allocated away from earlier treatment initiation towards this monitoring strategy. "Worse" value is indicated by a numerically higher ICER, and suggests that health benefits would be increased if resources were allocated towards earlier ARV initiation away from this monitoring strategy.

† WHO (World Health Organization) criteria for changing ARV regimen based on CD4 count

‡ Four strategies had ICERs that were not on the frontier but were sufficiently close to the frontier so that they were difficult to distinguish statistically. Three employed the conditional strategy "viral load only if CD4 meets WHO criteria" for: (1) frequency of 6 months and ARV switching threshold of 10,000 copies/mL [ICER > = $2200/QALY]; (2) frequency of 6 months and ARV switching threshold of 500 copies/mL [ICER > = $4900/QALY]; and (3) frequency of 3 months and ARV switching threshold of 10,000 copies/mL [ICER > = $6100/QALY. The fourth strategy was a CD4 alone strategy at a frequency of 12 months [ICER > = $5200/QALY).

¶ One strategy had an ICER that was not on the frontier but was sufficiently close to the frontier so that it was difficult to distinguish statistically: a CD4 alone strategy with a frequency of 6 months [ICER > = $6,500/QALY].

Results are only shown for strategies that maximized health benefits for some budget scenarios or willingness to pay for health benefits.

When willingness to pay remained limited to initiating cART at a CD4 count of 200 cells/mm^3 ^rather than 350 cells/mm^3^, the efficient frontier was mostly comprised of monitoring strategies that were structured conditionally, in which CD4 was obtained routinely and a follow-up viral load was only obtained if the CD4 count met WHO criteria for immunologic failure. Conservative monitoring frequencies (12 or six months rather than three months) and switching thresholds (10,000 copies/mL rather than 500 copies/mL) were preferred. In sensitivity analyses, only one strategy employing routine CD4 monitoring alone (every 12 months) was close to the efficient frontier.

As willingness to pay rose beyond initiating cART at a CD4 count of 350 cells/mm^3 ^rather than 200 cells/mm^3^, the efficient frontier continued to be mostly comprised of monitoring strategies that were structured conditionally; however, these strategies now employed less conservative monitoring frequencies and switching thresholds. Only one strategy employing routine CD4 monitoring alone (every six months) was close to the efficient frontier.

As willingness to pay greatly exceeded the value of earlier cART initiation, the efficient frontier became comprised of monitoring strategies that used routine viral load monitoring, with progressively greater frequencies and less conservative switching thresholds.

### Scenario with three available cART regimens

**Figure 3:**
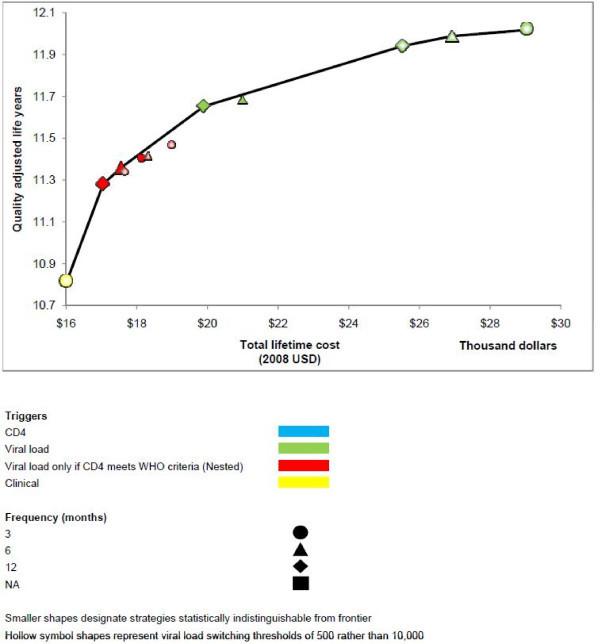
**Efficient frontier of HIV monitoring strategies assuming three cART regimens are available**.

When we explored a scenario in which three cART regimens were available (Table 3, Figure 3), strategies with routine CD4 monitoring alone continued to be excluded from the efficient frontier, and therefore never offered a good use of healthcare resources. As willingness to pay approached the value of cART initiation at a CD4 count of 350 cells/mm^3 ^rather than 200 cells/mm^3 ^(ICER $2600/QALY), the efficient frontier was comprised of monitoring strategies that were structured conditionally, where CD4 was obtained routinely and a follow-up viral load was only obtained if the CD4 count met WHO immunologic criteria. As willingness to pay greatly exceeded the value of earlier cART initiation, the efficient frontier was comprised of monitoring strategies that used routine viral load monitoring. In sensitivity analyses, no strategy employing routine CD4 monitoring alone was close to the efficient frontier.

**Table 3:** Value of alternative laboratory monitoring strategies compared to earlier treatment initiation, *assuming three antiretroviral (ARV) regimens are available*

Monitoring strategy	Freq-uency (mo.)	Viral load threshold for switching ARV (copies/ml)	5-year outcomes				Cost ($2008)	QALY	ICER ($/QALY)	Value compared with earlier treatment initiation*
							
			Mean # ARV rounds used	Mean new mut-ations	Median CD4 (cells/mm^3^)	Median HIV (log units)				
Clinical	3	N/A	1.32	1.02	271	2.65	16,017	10.814	N/A	N/A

Viral load only if CD4 meets WHO criteria†	12	10,000	1.3	1.1	270	2.70	17,050	11.281	2200	Similar

Viral load only if CD4 meets WHO criteria†‡	6	10,000	1.32	1.12	270	2.70	17,571	11.361	6500	Worse

Viral load	12	10,000	1.45	1.03	280	2.68	19,900	11.652	8000	Worse

Viral load¶	12	500	2.06	0.81	290	2.38	25,527	11.941	19,500	Worse

Viral load	6	500	2.12	0.77	290	2.36	26,927	11.988	29,800	Worse

Viral load	3	500	2.16	0.76	290	2.34	29,063	12.018	71,200	Worse

Mo.: months; QALY: quality-adjusted life year; ICER: incremental cost-effectiveness ratio.

* Earlier treatment initiation at CD4 of 350 cells/mm^3 ^compared with CD4 of 200 cells/mm^3^. "Better" value is indicated by a numerically lower ICER, and suggests that health benefits would be increased if resources were allocated away from earlier treatment initiation towards this monitoring strategy. "Worse" value is indicated by a numerically higher ICER, and suggests that health benefits would be increased if resources were allocated towards earlier ARV initiation away from this monitoring strategy.

† WHO (World Health Organization) criteria for changing ARV regimen based on CD4 count

‡ Four strategies had ICERs that were not on the frontier but were sufficiently close to the frontier so that they were difficult to distinguish statistically, all employing the conditional strategy, "viral load only if CD4 meets WHO criteria", for: (1) frequency of 12 months and ARV switching threshold of 500 copies/mL [ICER > = $3600/QALY]; (2) frequency of 6 months and ARV switching threshold of 500 copies/mL [ICER > = $5600/QALY]; (3) frequency of 3 months and ARV switching threshold of 10,000 copies/mL [ICER > = $5100/QALY; (4) frequency of 3 months and ARV switching threshold of 500 copies/mL [ICER > = $5100/QALY].

¶ One strategy had an ICER that was not on the frontier but was sufficiently close to the frontier so that it was difficult to distinguish statistically: viral load alone with a frequency of 6 months and switching threshold of 10,000 copies/mL [ICER > = $11,200/QALY].

Results are only shown for strategies that maximized health benefits for some budget scenarios or willingness to pay for health benefits.

### Scenario with variable number of cART regimens

**Figure 4:**
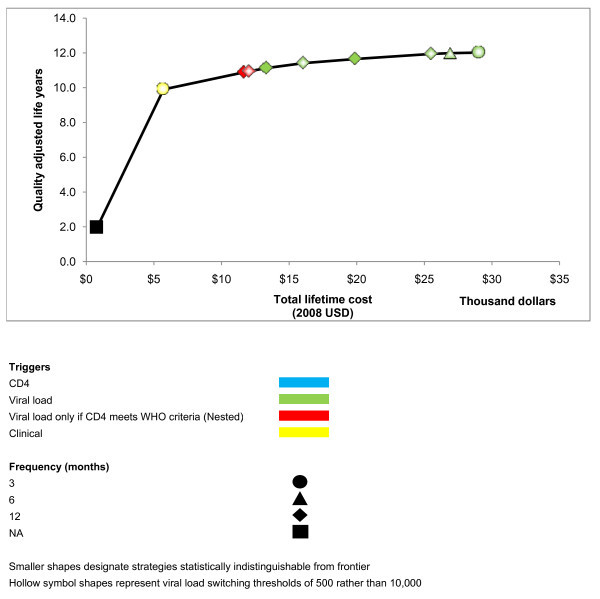
**Efficient frontier of HIV monitoring strategies assuming no fixed number of cART regimens available**.

When we considered a scenario that does not specify a fixed number of cART regimens, but rather allows the number of available cART regimens to vary (Table 4, Figure 4), strategies for routine CD4 monitoring alone continued to be excluded from the efficient frontier. At willingness-to-pay levels below that of earlier cART initiation, the efficient frontier was limited to a sole strategy (one cART regimen and using clinical rather than laboratory monitoring). As willingness-to-pay levels rose above that of early cART initiation, the greatest benefit was delivered by incorporating multiple regimens with routine viral load monitoring. In sensitivity analyses, only two strategies employing routine CD4 monitoring alone were close to the efficient frontier.

**Table 4:** Value of alternative laboratory monitoring strategies compared with earlier treatment initiation *without any fixed assumption about numbers of available antiretroviral (ARV) regimens*

# ARV regimens	Monitoring strategy	Freq-uency (mo.)	Viral load thres-hold	5-year outcomes				Cost, $2008	QALY	ICER, $/QALY	Value com-pared with earlier treatment initiation*
								
				Mean # ARV rounds used	Mean new mut-ations	Median CD4 (cells/mm^3^)	Median HIV (log units)				
0	Nothing	NA	NA	0	0	0	0	807	1.966	NA	NA

1	Clinical	3	NA	1	1.05	265	2.79	5713	9.901	600	Better

2	Viral load only if CD4 meets WHO criteria†‡	12	10,000	1.23	1.09	270	2.7	11,691	10.890	6000	Worse

2	Viral load only if CD4 meets WHO criteria†¶	12	500	1.27	1.06	270	2.66	12,060	10.948	6400	Worse

2	Viral load	12	10,000	1.33	1.02	277	2.69	13,308	11.125	7100	Worse

2	Viral load§	12	500	1.67	0.82	285	2.42	16,035	11.412	9500	Worse

3	Viral load	12	10,000	1.45	1.03	280	2.68	19,900	11.652	16,100	Worse

3	Viral load	12	500	2.06	0.81	290	2.38	25,527	11.941	19,500	Worse

3	Viral load	6	500	2.12	0.77	290	2.36	26,927	11.988	29,800	Worse

3	Viral load	3	500	2.16	0.76	290	2.34	29,063	12.018	71,200	Worse

Mo.: months; QALY: quality-adjusted life year; ICER: incremental cost-effectiveness ratio.

* Earlier treatment initiation at CD4 of 350 cells/mm^3 ^compared with CD4 of 200 cells/mm^3^. "Better" value is indicated by a numerically lower ICER, and suggests that health benefits would be increased if resources were allocated away from earlier treatment initiation towards this monitoring strategy. "Worse" value is indicated by a numerically higher ICER, and suggests that health benefits would be increased if resources were allocated towards earlier ARV initiation away from this monitoring strategy.

† WHO (World Health Organization) criteria for changing ARV regimen based on CD4 count

‡ Three strategies had ICERs that were not on the frontier but were sufficiently close to the frontier so that they were difficult to distinguish statistically, all allowing 2 ARV regimens. Two employed the conditional strategy, "viral load only if CD4 meets WHO criteria", for: (1) frequency of 6 months and ARV switching threshold of 10,000 copies/mL [ICER > = $2200/QALY]; (2) frequency of 6 months and ARV switching threshold of 500 copies/mL [ICER > = $4900/QALY]. The third employed a CD4 alone strategy with a frequency of 12 months [ICER > = $5200/QALY].

¶ Two strategies had an ICER that was not on the frontier but was sufficiently close to the frontier so that it was difficult to distinguish statistically, both allowing 2 ARV regimens. One employed the strategy, "viral load only if CD4 meets WHO criteria", with frequency of 3 months and ARV switching threshold of 10,000 copies/mL [ICER > = $6100/QALY] and the other was a CD4 alone strategy with a frequency of 6 months [ICER > = $6400/QALY],

§ Two strategies had an ICER that was not on the frontier but was sufficiently close to the frontier so that it was difficult to distinguish statistically, both employing viral loads alone with 6 month frequency, the first using an ARV switching threshold of 500 copies/mL and allowing 2 ARV regimens [ICER > = $13,900/QALY] and the second using an ARV switching threshold of 10,000 copies/mL and allowing 3 ARV regimens [ICER > = $14,900/QALY].

Results are only shown for strategies that maximized health benefits for some budget scenarios or willingness to pay for health benefits.

Conditional strategies were no longer on the efficient frontier because the ICER of routinely offering multiple cART regimens (compared with providing one cART regimen only) was fairly high (ICER > $5000/QALY), and because the ICER of any laboratory testing strategy would only be favourable if multiple cART regimens were routinely offered. As willingness to pay exceeded the ICER of offering multiple cART regimens, they were also high enough to support the ICER of routine use of viral load testing.

### Sensitivity analyses

Sensitivity analyses suggested that efficient frontiers were robust to alternative assumptions (Additional file 1, Figure S2), with monitoring strategies based on CD4 counts alone almost never falling on the efficient frontier. A notable exception to this stability occurred when assumptions were varied regarding the pricing of second- and third-line cART regimens relative to first-line cART. When later cART regimens were assumed to be no more expensive than first-line cART regimens, the value of monitoring strategies that involved routine viral load testing became more favourable.

## Discussion

Our results have several implications for monitoring of HIV-infected patients in resource-limited settings. First, routine CD4 monitoring alone is unlikely to be a preferred strategy, regardless of available resources, willingness to pay or availability of treatment options. This is likely attributable to the poor sensitivity and specificity of CD4 testing for detecting treatment failure and viral rebound [19]. Because routine CD4 monitoring alone (e.g., without viral load to confirm treatment failure) is never preferred, our results suggest that the WHO recommendation to use viral load to confirm treatment failure should not be diluted with the phrase, "where resources are available", and instead should employ the same strength of language that it applies to earlier cART initiation. Indeed, employing CD4 counts together with conditional viral load testing is a preferred strategy under a wide range of willingness-to-pay and treatment availability scenarios.

**Figure 5:**
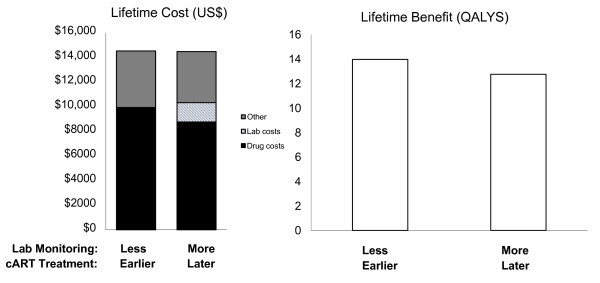
**Comparison of alternative strategies for allocating expenditures for a hypothetical HIV patient in East Africa**. This figure shows a comparison of monitoring strategies for a patient newly diagnosed with HIV with a CD4 count of 350 cells/mm^3^. The right pair of bars shows a strategy that relies on routine viral load monitoring, whereas the left set of bars shows a strategy that relies more on clinical monitoring, and reallocates the money saved on less laboratory monitoring to fund earlier initiation of ARV. Even though both strategies incur the same lifetime expenditures, the strategy that employs less laboratory monitoring to enable earlier ARV initiation increases life expectancy by 1.5 quality-adjusted life years.

Second, routine viral load testing alone is only a preferred strategy at levels of willingness to pay that far exceed those of earlier cART initiation. In other words, our results suggest that a programme routinely monitoring viral loads, but starting cART at a CD4 of 200 cells/mm^3 ^rather than 350 cells/mm^3^, will save more high-quality years of life if it reallocates some laboratory expenditures towards drugs allowing for earlier initiation of cART (Figure 5). These results suggest that the WHO conditional recommendation to use viral load testing every six months to check for viral replication "where routinely available" should be interpreted, more concretely, as meaning "in those settings where all patients are already started on cART at a CD4 count of 350 cells/mm^3 ^(rather than 200 cells/mm^3^)".

Third, when monitoring includes viral load in resource-limited settings, the switching threshold conferring the greatest value is more likely to be 10,000 copies/mL than lower thresholds, and raises the question of whether the recent change in threshold advocated by WHO (5000 copies/mL rather than 10,000 copies/mL) is a step in the right direction, especially when the downstream cost burdens of switching first-line regimens to far more expensive, second-line regimens might make it more difficult to simultaneously adhere to other costly changes in its recommendations.

Fourth, if programmes are considering alternative monitoring strategies at the same time that they are weighing how many cART regimen options to offer, our results suggest that they can save more high-quality years of life by routinely offering fewer regimens with less intense monitoring strategies, and by reallocating saved resources on earlier initiation of cART.

Fifth, the bulk of expenditures from routine viral load testing did not arise from the cost of the viral load test itself, but rather originated from the downstream costs of more frequent switches to expensive second- and third-line regimens. When later cART regimens were assumed to be no more expensive than first-line cART regimens, monitoring strategies that involved routine viral load testing became more favourable. These results suggest that even if viral load tests become cheaper, they may not offer favourable value if there is no change in the relative pricing of different regimens. In contrast, if later cART regimens become less expensive relative to first-line regimens, viral load tests may offer favourable value even if the tests themselves remain expensive.

Our estimates for the ICER of cART ($600/QALY) were very similar to other published analyses ($590 per life year, Goldie; $628/QALY, Bishai) [10,11]. Like other analyses, the incremental cost effectiveness of routine CD4 testing was unfavourable compared with some alternative resource uses [5]. Our estimates for the ICER of viral load testing are difficult to compare with other published analyses because we considered conditional strategies, in which viral load is not ordered routinely. Still, our results are concordant with other analyses suggesting that lives would be saved by allocating resources away from routine viral load testing and towards other resource-constrained care strategies (e.g., earlier initiation of cART) [5].

Our analyses have notable limitations. Our simulation is not a transmission model, and therefore does not consider how more conservative monitoring strategies might lead to: (1) delayed detection of antiretroviral resistance and its spread; and (2) higher viral loads in treated patients, which have been associated with increased transmission rates. However, this consideration is unlikely to alter inferences for decision making because the increase in resistance accumulation is likely to be modest (less than one resistance mutation over a five-year period) (Tables 2, 3, 4).

Furthermore, allocating funds towards earlier treatment initiation would have a far more profound effect on viral load (and subsequently on HIV transmission risk) because people who are treated earlier will have low viral loads for a longer portion of the time they are infected. In addition, while the simulation is sufficiently detailed to represent clinical differences among subtypes of nucleoside reverse transcriptase inhibitors (e.g., inducing non-thymidine-analogue mutations versus inducing thymidine anologue mutations), it is not sufficiently detailed to represent clinical differences among individual drugs within each subtype (e.g., zidovudine versus stavudine).

A distinctive strength of our work is that we evaluated a broad matrix of monitoring options, including some strategies that are not guideline recommended at the current time, but which might seem like plausible alternatives to some decision makers (for example, obtaining routine viral load without routine CD4 counts). Indeed, the ability to simultaneously evaluate a broad range of monitoring options beyond those currently employed is one of the key methodological strengths of using mathematical modelling in general, and of the current report in particular.

## Conclusions

In conclusion, our computer simulation suggests that shifting resources away from routine laboratory monitoring and toward earlier initiation of cART has the potential to increase the number of lives saved with HIV treatment in a resource-constrained environment.

## Funding

This work is supported by National Institute of Allergy and Infectious Disease Award UO1AI069911-01 (IeDEA East Africa), US National Institutes of Health. MCB is employed by the US NIH, which provided funding for this study through a grant. The study sponsor had no role in the study design, interpretation of data, the writing of the paper, or the decision to submit the paper for publication.

## Competing interests

The authors declare that they have no competing interests.

## Authors' contributions

RSB designed the study concept. RSB and KN developed the model and KN programmed the model. LD, CTY, BM, SK, MCB and KWK acquired and/or interpreted the data. RSB analyzed results and wrote the manuscript. RSB, CTY, LD, MCB and KWK revised the manuscript. All authors read and approved the final manuscript.

## Supplementary Material

Additional file 1 **Appendix**. The Appendix describes in detail the methods underlying the revision of the model and its calibration. The Appendix figures show results of model calibration and results of sensitivity analyses [26-29]. Click here for file

## References

[B1] Calmy A Ford N Hirschel B Reynolds SJ Lynen L Goemaere E Garcia de la Vega F Perrin L Rodriguez W HIV viral load monitoring in resource-limited regions: optional or necessary? Clin Infect Dis 2007 14 128 134 10.1086/510073 17143828

[B2] Elliott JH Lynen L Calmy A De Luca A Shafer RW Zolfo M Clotet B Huffam S Boucher CA Cooper DA Schapiro JM Rational use of antiretroviral therapy in low-income and middle-income countries: optimizing regimen sequencing and switching AIDS 2008 14 2053 2067 10.1097/QAD.0b013e328309520d 18753937

[B3] Fowler MG Owor M Monitoring HIV treatment in resource-limited settings: reassuring news on the usefulness of CD4(+) cell counts J Infect Dis 2009 14 1255 1257 10.1086/597618 19317627

[B4] Johannessen A Garrido C Zahonero N Sandvik L Naman E Kivuyo SL Kasubi MJ Gundersen SG Bruun JN de Mendoza C Dried blood spots perform well in viral load monitoring of patients who receive antiretroviral treatment in rural Tanzania Clin Infect Dis 2009 14 976 981 10.1086/605502 19663598

[B5] Phillips AN Pillay D Miners AH Bennett DE Gilks CF Lundgren JD Outcomes from monitoring of patients on antiretroviral therapy in resource-limited settings with viral load, CD4 cell count, or clinical observation alone: a computer simulation model Lancet 2008 14 1443 1451 10.1016/S0140-6736(08)60624-8 18440426

[B6] Schooley RT Viral load testing in resource-limited settings Clin Infect Dis 2007 14 139 140 10.1086/510090 17143830

[B7] WHO Antiretroviral therapy for HIV infection in adults and adolescents: Recommendations for a public health approach: 2010 revision http://www.who.int/hiv/pub/arv/adult2010/en/index.html 23741771

[B8] ART-LINC of IeDEA Study Group Keiser O Tweya H Boulle A Braitstein P Schecter M Brinkhof MW Dabis F Tuboi S Sprinz E Pujades-Rodriguez M Calmy A Kumarasamy N Nash D Jahn A MacPhail P Luthy R Wood R Egger M Switching to second-line antiretroviral therapy in resource-limited settings: comparison of programmes with and without viral load monitoring AIDS 2009 14 1867 1874 19531928 10.1097/QAD.0b013e32832e05b2 PMC2956749

[B9] DART Trial Team Mugyenyi P Walker AS Hakim J Munderi P Gibb DM Kityo C Reid A Grosskurth H Darbyshire JH Ssali F Bray D Katabira E Babiker AG Gilks CF Grosskurth H Munderi P Kabuye G Nsibambi D Kasirye R Zalwango E Nakazibwe M Kikaire B Nassuna G Massa R Routine versus clinically driven laboratory monitoring of HIV antiretroviral therapy in Africa (DART): a randomised non-inferiority trial Lancet 2010 14 123 131 20004464 10.1016/S0140-6736(09)62067-5 PMC2805723

[B10] Goldie SJ Yazdanpanah Y Losina E Weinstein MC Anglaret X Walensky RP Hsu HE Kimmel A Holmes C Kaplan JE Freedberg KA Cost-effectiveness of HIV treatment in resource-poor settings--the case of Cote d'Ivoire N Engl J Med 2006 14 1141 1153 10.1056/NEJMsa060247 16971720

[B11] Bishai D Colchero A Durack DT The cost effectiveness of antiretroviral treatment strategies in resource-limited settings AIDS 2007 14 1333 1340 10.1097/QAD.0b013e328137709e 17545710

[B12] Braithwaite RS Justice AC Chang CC Fusco JS Raffanti SR Wong JB Roberts MS Estimating the proportion of patients infected with HIV who will die of comorbid diseases Am J Med 2005 14 890 898 10.1016/j.amjmed.2004.12.034 16084183

[B13] Braithwaite RS Roberts MS Chang CC Goetz MB Gibert CL Rodriguez-Barradas MC Shechter S Schaefer A Nucifora K Koppenhaver R Justice AC Influence of alternative thresholds for initiating HIV treatment on quality-adjusted life expectancy: a decision model Ann Intern Med 2008 14 178 185 18252681 10.7326/0003-4819-148-3-200802050-00004 PMC3124094

[B14] Braithwaite RS Shechter S Chang CC Schaefer A Roberts MS Estimating the rate of accumulating drug resistance mutations in the HIV genome Value Health 2007 14 204 213 10.1111/j.1524-4733.2007.00170.x 17532813

[B15] Braithwaite RS Shechter S Roberts MS Schaefer A Bangsberg DR Harrigan PR Justice AC Explaining variability in the relationship between antiretroviral adherence and HIV mutation accumulation J Antimicrob Chemother 2006 14 1036 1043 10.1093/jac/dkl386 17023498

[B16] Geng EH Bangsberg DR Musinguzi N Emenyonu N Bwana MB Yiannoutsos CT Glidden DV Deeks SG Martin JN Understanding reasons for and outcomes of patients lost to follow-up in antiretroviral therapy programs in Africa through a sampling-based approach J Acquir Immune Defic Syndr 2010 14 405 411 10.1097/QAI.0b013e3181b843f0 19745753 PMC3606953

[B17] Markowitz H Portfolio Selection J Finance 1952 14 405 411

[B18] Gold MR Seigel JE Russell LB Weinstein MC Cost-Effectiveness in Health and Medicine 1996 New York: Oxford University Press

[B19] Mee P Fielding KL Charalambous S Churchyard GJ Grant AD Evaluation of the WHO criteria for antiretroviral treatment failure among adults in South Africa AIDS 2008 14 1971 1977 10.1097/QAD.0b013e32830e4cd8 18784460

[B20] World Health Organization WHO case definitions of HIV for surveillance and revised clinical staging and immunologic classification of HIV-related disease in adults and children http://www.who.int/hiv/pub/guidelines/hivstaging/en/index.html

[B21] Johnson VA Brun-Vezinet F Clotet B Gunthard HF Kuritzkes DR Pillay D Schapiro JM Richman DD Update of the drug resistance mutations in HIV-1: December 2009 Top HIV Med 2009 14 138 145 20068260

[B22] Braithwaite RS Kozal MJ Chang CC Roberts MS Fultz SL Goetz MB Gibert C Rodriguez-Barradas M Mole L Justice AC Adherence, virological and immunological outcomes for HIV-infected veterans starting combination antiretroviral therapies AIDS 2007 14 1579 1589 10.1097/QAD.0b013e3281532b31 17630553 PMC3460378

[B23] Braithwaite RS Goulet J Kudel I Tsevat J Justice AC Quantifying the decrement in utility from perceived side effects of combination antiretroviral therapies in patients with HIV Value Health 2008 14 975 979 10.1111/j.1524-4733.2007.00315.x 18225989 PMC3121315

[B24] Freedberg KA Scharfstein JA Seage GR Losina E Weinstein MC Craven DE Paltiel AD The cost-effectiveness of preventing AIDS-related opportunistic infections JAMA 1998 14 130 136 10.1001/jama.279.2.130 9440663

[B25] Red Book. Pharmacy's Fundamental Reference 2009 113 PDR Network

[B26] World Health Statistics http://www.who.int/whosis/whostat/EN_WHS08_Full.pdf

[B27] Mills EJ Nachega JB Buchan I Orbinski J Attaran A Singh S Adherence to antiretroviral therapy in sub-Saharan Africa and North America: a meta-analysis JAMA 2006 14 679 690 10.1001/jama.296.6.679 16896111

[B28] Yiannoutsos CT An MW Frangakis CE Musick BS Braitstein P Wools-Kaloustian K Sampling-based approaches to improve estimation of mortality among patient dropouts: experience from a large PEPFAR-funded program in Western Kenya PLoS One 2008 14 e3843 10.1371/journal.pone.0003843 19048109 PMC2585792

[B29] Justice AC Dombrowski E Conigliaro J Fultz SL Gibson D Madenwald T Veterans Aging Cohort Study (VACS): Overview and description Med Care 2006 14 S13 24 10.1097/01.mlr.0000223741.02074.66 16849964 PMC3049942

