# Supplementary material for: Alternative antiretroviral monitoring strategies for HIV-infected patients in east Africa: opportunities to save more lives?
Source: J Int AIDS Soc. 2011 Jul 30;14:38. doi: 10.1186/1758-2652-14-38 (PMC3163507; doi:10.1186/1758-2652-14-38)
Supplement: Additional file 1 — Appendix. The Appendix describes in detail the methods underlying the revision of the model and its calibration. The Appendix figures show results of model calibration and results of sensitivity analyses [26-29]. [file 1758-2652-14-38-S1.DOC]

**Additional file 1.**

**Alternative antiretroviral monitoring strategies for HIV-infected patients in east Africa: opportunities to save more lives?**

R Scott Braithwaite, *et al.*

**APPENDIX**

We have created an updated version of our prior published HIV computer simulation (referred henceforth as “version 2.0”). This simulation has been updated (1) to allow specification of a wide variety of possible monitoring strategies, (2) to allow calibration using data from resource-limited settings, and (3) to consider a specifiable number of cART regimens or a specifiable number of drugs within each cART category (and can “run out” of regimens when intolerance and/or resistance has developed to all).

**Monitoring Strategies**

Version 2.0 allows a flexible specification of monitoring strategies in 1 or more domains: clinical (e.g., surveillance for AIDS-defining events), immunologic (e.g., CD4 testing), and virologic (e.g., viral load testing). For strategies in which CD4 testing is involved, cART switching criteria was assumed to follow WHO criteria (e.g., persistent CD4 count <100 cells/mm^3^, decline of more than 50% from highest level, or failure to increase above pre-treatment CD4 count) [20]. For strategies in which viral load is involved, the threshold could be set to variable levels (e.g., 500, 1000, 5000 or 10,000 copies/ml). The frequency of monitoring in these different domains (e.g., 3 months, 6 months, or 12 months) and their interdependency can be varied. For example, a monitoring strategy can consist of measuring CD4 every 6 months with clinical assessments every 3 months per WHO criteria and viral load assessed every 6 months only if CD4 criteria are positive. Alternatively, a monitoring strategy could be far simpler, consisting of routine viral load monitoring every 3 months alone.

**Calibration**

We aimed to calibrate version 2.0 of the simulation separately, for two different settings, one in a resource-poor environment and one in a resource-rich environment. We selected east Africa as our resource-poor environment of interest, and we selected North America as our resource-rich area of interest. While we realized that some inputs to the simulation would necessarily be different between these two settings; we aimed for a parsimonious calibration procedure that would avoid overfitting the simulation to any one setting, and that would incorporate as few differences in model inputs as would be possible, beyond those known to be substantially different. In addition to the pragmatic motivation of avoiding an overfitted and less generalizable model, we also had a theoretical motivation for our parsimonious calibration procedure: differences in outcomes between resource-rich and resource-poor settings are more likely to be attributable to differences in care settings (which generally correspond to inputs to the simulation) rather than differences in HIV or host biological characteristics (which generally correspond to structural features of the simulation).

Accordingly, whenever plausible, non-cost data inputs were unchanged from resource-rich settings (Manuscript Table 1) [21-25]. However, inputs were varied when they were likely to be different for east African populations (e.g., non-HIV-related mortality, cART intolerance, and cART adherence). We calculated age- and sex-specific non-HIV-related mortality from lifetables of Kenya mortality data, incorporating a multiplicative term [allowed to vary during calibration] to compensate for bias that could result because individuals with HIV may have comorbidities, risk behaviors, and other factors that elevate their mortality beyond those of age and sex-matched persons in the general population; and for bias resulting from potentially double-counting HIV mortality (since a substantial proportion of mortality in Kenya is from HIV) [26]. In an analogous fashion, because tuberculosis may amplify HIV-related mortality disproportionately in resource-limited settings, we added a multiplicative term that enabled HIV-related mortality to fluctuate. Intolerance (the rate of regimen change due to side effects rather than failure) is lower in resource-limited settings because of fewer treatment alternatives and cART adherence is better in resource-limited settings. We allowed these terms to vary during calibration, and we selected the input values that resulted in the best fit between simulation output and clinical data [27].

We calibrated the simulation for east Africa by comparing its results with clinical data obtained from the United States Agency for International Development Partnership with the Academic Model Providing Access to Healthcare (USAID-AMPATH) partnership, using a pre-specified battery of comparisons based on our previous calibration methods [12-15]. In particular, our pre-specified calibration criteria involved survival, time to switching of cART regimen 1, time to switching of cART regimen 2, and mean CD4 count; where “switching” was defined as changing 2 or more cART drugs. We used statistical methods, previously published by our group, to adjust passively assessed patient mortality by employing random sampling of patients lost to follow-up with active surveillance and estimating overall mortality by combining the data with patients who remained in follow-up [28]. In addition, we estimated the longitudinal trajectories of CD4 counts based on data obtained in the subgroup of USAID-AMPATH patients retained in clinic. Unfortunately, the later data are potentially biased in unpredictable ways (for example, tracking median CD4 count over time might overestimate the true CD4 trajectory as patients with lower CD4 counts may die or be lost-to-follow-up at higher rates than patients with higher CD4 counts). For this reason, we placed the greatest emphasis on ensuring that the survival curves, produced by our simulation model, fit the clinical data well.

We calibrated the simulation for North America by comparing its results with clinical data obtained from the Veterans Aging Cohort Study (VACS) [29], using the same pre-specified battery of comparisons based on our previous calibration methods [12-15].

During calibration, we explored three alternative methods to improve goodness of fit: minimizing mean-squared differences, minimizing the Kolmogorov-Smirnov distance statistic and visual inspection. Ultimately, we chose to use visual inspection because the survival curves did not fit closely enough using the other two more quantitative approaches. While it may have been possible to use one of these more formal approaches if the individual calibration criteria were assigned relative valuation “weights” (e.g., with survival valued much more than the others), we did not wish to formally weight the calibration criteria because we did not have a priori criteria for establishing such relative weights.

When calibrating version 2.0 for east Africa, we found that simulation versus clinical data were extremely close for survival (Figure S1A). They were similar for the later phase of time to treatment switching for regimen 1 (Figure S1B), and for time to treatment switching for regimen 2 (Figure S1C). We found that improving the fit for the early phase of regimen 1 compromised fit elsewhere, likely because the simulation could only apply WHO CD4 criteria for switching cART in a binary fashion, whereas in clinical care, WHO CD4 criteria may be applied with increasing stringency over the early phase of a regimen. We decided to accept this minor loss of fidelity as a worthwhile tradeoff to avoid making the simulation and the variety of monitoring options even more complex. We found that increase in CD4 counts over time was lower than observed in AMPATH (Figure S1D), but was similar to that observed in less biased data (for example, mean CD4 count at 5 years was increased in DART by between 275 and 298 cells/mm^3^ depending on aggressiveness of monitoring [9]; CD4 counts in our simulation was increased by between 265 and 290 cells/mm^3^ at 5 years depending on aggressiveness of monitoring); and we did not strive for better goodness of fit because of the potential danger of overfitting the simulation to a biased sample. When calibrating version 2.0 for North America, we found that simulation results were able to come substantially close to clinical results for survival, time to treatment switching for cART regimen 1, time to treatment switching for cART regimen 2, and CD4 elevation (Figure S1).

**Flexible specification of number of cART drugs or regimens**

Version 2.0 enables model users to specify a number of available drugs in each cART category. The number of distinct possible regimens then becomes an emergent property of the simulation, based on cross-resistance patterns within classes and between classes; and regimen choices are made by the model based on number of available drugs and resistance. This logic is suitable for resource-rich areas, where resistance testing might be a part of routine care. Alternatively, model users can elect not to specify a number of available drugs in each cART category, but instead to specify fixed numbers and sequences of regimen types. This logic is suitable for resource-limited areas, where resistance testing is unlikely to be a part of routine care. For our North America calibration, we used the logic in which regimens choices are based on number of available drugs and resistance patterns, since in resource rich areas resistance patterns be a regular part of care. For our east Africa calibration and subsequent analyses, we used the logic in which a fixed number and sequence of regimens was designated. (While one could argue that presence or absence of resistance testing should itself be included in the broad matrix of alternative monitoring strategies for resource-limited settings, we decided not to pursue this idea in the current analyses.)

Of note, the simulation does not have the granularity to distinguish between different nucleoside reverse transcriptase inhibitors that induce thymidine analogue mutations, and therefore does not distinguish between zidovudine and stavudine. However, in sensitivity analyses, we vary assumptions about cART toxicity across a plausible range that encompasses the toxicity profiles of different thymidine analogue reverse transcriptase inhibitors, so their differential toxicity is unlikely to impact the validity of our results.

**Figure S1. Calibration of survival (A), time to 1^st^ cART regimen switch (B), time to 2^nd^ cART regimen switch (C), and CD4 elevation (D).** Simulation results are compared to clinical data from large cohorts in east Africa and North America.

S1A.

S1B.

S1C.

S1D.

**Figure S2. Sensitivity analysis of how the cost-effectiveness of monitoring strategies is impacted by alternative model assumptions.** An “X” indicates a strategy that is on the efficient frontier. Grey backgrounds indicate strategies that are on the efficient frontier in base case analysis. Black outlines signify strategies that confer cost-effectiveness more favorable than that of initiating cART at a CD4 count of 350 cells/mm^3^ rather than 200 cells/mm^3^. Efficient frontiers are fairly robust to different assumptions (e.g., most “X”s fall within the grey columns), with the notable exception of when the cost of 2^nd^ and 3^rd^ line cART regimens is assumed to be similar to the cost of 1^st^ line cART, which causes more monitoring strategies to fall on the efficient frontier, with cost-effectiveness more favorable than that of earlier cART initiation. Assuming lesser virologic suppression from cART under ideal circumstances or assuming lesser cross-resistance also impacts which strategies fall on the efficient frontier, but does not cause any strategies to have more favorable cost-effectiveness than that of earlier cART initiation.
